# Competency of Amphibians and Reptiles and Their Potential Role as Reservoir Hosts for Rift Valley Fever Virus

**DOI:** 10.3390/v12111206

**Published:** 2020-10-23

**Authors:** Melanie Rissmann, Nils Kley, Reiner Ulrich, Franziska Stoek, Anne Balkema-Buschmann, Martin Eiden, Martin H. Groschup

**Affiliations:** 1 Institute of Novel and Emerging Infectious Diseases, Friedrich-Loeffler-Institut, 17493 Greifswald-Insel Riems, Germany; melanie.rissmann@fli.de (M.R.); nilskley@yahoo.com (N.K.); franziska.stoek@fli.de (F.S.); anne.balkema-buschmann@fli.de (A.B.-B.); martin.eiden@fli.de (M.E.); 2 Department of Experimental Animal Facilities and Biorisk Management, Friedrich-Loeffler-Institut, 17493 Greifswald-Insel Riems, Germany; reiner.ulrich@vetmed.uni-leipzig.de; 3 Institute of Veterinary Pathology, Leipzig University, 04103 Leipzig, Germany

**Keywords:** Rift Valley fever virus, reservoir, amphibians, reptiles, experimental infection

## Abstract

Rift Valley fever phlebovirus (RVFV) is an arthropod-borne zoonotic pathogen, which is endemic in Africa, causing large epidemics, characterized by severe diseases in ruminants but also in humans. As in vitro and field investigations proposed amphibians and reptiles to potentially play a role in the enzootic amplification of the virus, we experimentally infected African common toads and common agamas with two RVFV strains. Lymph or sera, as well as oral, cutaneous and anal swabs were collected from the challenged animals to investigate seroconversion, viremia and virus shedding. Furthermore, groups of animals were euthanized 3, 10 and 21 days post-infection (dpi) to examine viral loads in different tissues during the infection. Our data show for the first time that toads are refractory to RVFV infection, showing neither seroconversion, viremia, shedding nor tissue manifestation. In contrast, all agamas challenged with the RVFV strain ZH501 carried virus genomes in the spleens at 3 dpi, but the animals displayed neither viremia nor virus shedding. In conclusion, the results of this study indicate that amphibians are not susceptible and reptiles are only susceptible to a low extent to RVFV, indicating that both species play, if at all, rather a subordinate role in the RVF virus ecology.

## 1. Introduction

Rift Valley fever phlebovirus (RVFV) is an arthropod-borne (arbo) zoonotic virus that is endemic throughout extensive parts of Africa and causes substantial recurrent outbreaks in Africa and the Arabian Peninsula. RVFV is an RNA virus of the family *Phenuiviridae*, genus Phlebovirus, with a tripartite ambisense genome, compromising a large (L), medium (M) and a small (S) segment. The L segment encodes the RNA-dependent RNA polymerase. The M segment encodes for the glycoproteins Gn and Gc and for a nonstructural protein NSm. The S segment encodes the nucleoprotein NP and the nonstructural protein NSs [1].

RVFV has been found in more than 30 mosquito species, of which *Aedes* spp. comprise the primary vectors for transmission and spread [2]. Transovarial transmissions of RVFV in *Aedes* spp. can promote the maintenance of the virus during enzootic periods [3]. Ecological factors (such as massive precipitations and inundations) can favor the proliferation of infected and non-infected mosquito vectors and this abundance can subsequently drive RVFV epidemic cycles [2]. The virus has a significant impact on local socio-economy and health systems with recurrent and significant outbreaks in many parts of Africa and on the Arabian Peninsula [4]. Clinical manifestations of Rift Valley fever (RVF) in livestock are species- and age-dependent. While most adult ruminants show only mild symptoms such as fever and apathy, so-called “abortion storms” with fetal and newborn fatalities of up to 100% are frequently observed in small ruminants [5]. Humans are infected primarily via contact to viremic animals and to their infectious blood and tissues [5]. Most human infections cause only mild, flu-like symptoms. However, 1–2% of cases are characterized by severe meningoencephalitis, retinitis or even hemorrhagic fever [6,7].

While the transovarial transmission of RVFV within *Aedes* spp. is known to favor the endemic maintenance of the virus, the presence of a yet unknown reservoir host has been repeatedly hypothesized [8,9,10,11,12,13]. Reservoir hosts would beneficially compensate suboptimal conditions for vector abundance. As previously defined, an optimal arboviral reservoir host is susceptible to infection and develops an extensive long-lasting viremia without showing severe symptoms. The reservoir population itself should be actively reproducing and a frequent contact between the vector and the reservoir host should be granted [14]. Olive et al. reviewed the potential role of several wild mammals to act as a reservoir host for RVFV. Serological and/or virological evidence of RVFV infections was revealed for 35 wild mammal species, however, their role in the transmission and maintenance of RVFV remains enigmatic. So far, the data are too fragmented or contradictory to define their role as RVFV reservoir hosts [13].

Amphibians and reptiles could represent potential hosts for arboviruses, as they make up a large component of the vertebrate biomass in terrestrial biological systems and inhabit wetland areas that are ideal breeding grounds for mosquitoes [15,16]. Indeed, various authors have recurrently reported evidence of amphibians and reptiles playing a role in the ecology of diverse arboviruses. Amphibians and reptiles play a role in the infection cycle of Alphaviruses (e.g., Western equine encephalitis virus [17,18,19,20,21] and Eastern equine encephalitis virus [22,23,24,25]), as shown by field studies (mosquito blood meal analyses) and by experimental infections. Experimental evidence of replication of Chikungunya virus was also found in amphibians and reptiles [26]. Additionally, evidence of Flaviviruses (e.g., West Nile virus [27,28,29,30,31,32,33] and Japanese encephalitis virus [34,35,36]) was found in several studies in amphibians and reptiles. Recently, it was shown that frogs can be experimentally infected with Zika virus, although the magnitude of viremia was low [37]. Furthermore, an Orthobunyavirus was isolated from the blood of a Texas soft-shelled turtle (*Apalone spinifera emoryi*) in the United States [38].

Since the initial RVFV description in 1931, when tortoises and frogs were experimentally infected, amphibians and reptiles were generally assumed to be non-susceptible to RVFV [39]. However, in 2006–2007, an investigation in Kenya observed a proportion of RVFV-infected mosquitoes to feed on frogs [40]. Additionally, another study found *Culex neavei* and *Mansonia uniformis* from Uganda to feed on amphibians and reptiles [41]. Both of these mosquito species had been considered to be naturally infected with RVFV in earlier reports [2]. Furthermore, recent in vitro studies demonstrated that cells of the African clawed frog (*Xenopus laevis*) are susceptible to RVFV [42]. Consequently, amphibians and reptiles have been proposed to be involved in the RVFV infection cycle.

To evaluate the potential of amphibians and reptiles as reservoirs for the maintenance of RVFV, African common toads (*Amietophrynus regularis*) and common agamas (*Agama agama*) were experimentally infected with the attenuated RVFV vaccine strain MP-12 and the highly virulent ZH501 strain. A limited replication of MP-12 was repeatedly demonstrated in different hosts [43,44,45], thus the infection with this strain was chosen to determine the ability of amphibians and reptiles to replicate even low pathogenic virus variants. Additionally, the infection with MP-12 was used to evaluate and set up the experimental conditions with a biosafety level 2 agent. ZH501 was chosen as a highly pathogenic RVFV strain, with documented significant manifestation in susceptible hosts. Their overall susceptibility, viremia and virus shedding as well as the RVFV tissue manifestation patterns at different time points after infection were examined.

## 2. Materials and Methods

### 2.1. Virus and Cell Culture

The RVFV MP-12 strain was kindly provided by Richard Elliot, University of Glasgow, Centre for virus research, United Kingdom. MP-12 was propagated in Vero 76 cells (Collection of Cell Lines in Veterinary Medicine CCLV, Friedrich-Loeffler-Institut, Germany). The ZH501 strain of RVFV was kindly provided by Jeroen Kortekaas, University of Wageningen, Wageningen Bioveterinary Research Subdivision Virology, The Netherlands. ZH501 was propagated in Vero E6 cells (CCLV). The infectivity of the applied virus strain and specific stock was previously confirmed, since infection of mice (65 infected mice) and sheep (8 infected sheep) resulted in classical clinical manifestation and partially in mortalities in mice (manuscript in preparation).

Virus titers were determined using 50% Tissue Culture Infective Dose (TCID_50_) assays, calculated as described by Spearman and Kaerber. Briefly, 100 µL of 10-fold serial virus dilutions was added to 90% confluent cell monolayers in 96-well plates. After incubation at 37 °C, 5% CO_2_ for 6 days, plates were fixed with neutral buffered formalin and stained with 1% crystal violet.

### 2.2. Animals and Experimental Design

**Figure 1:**
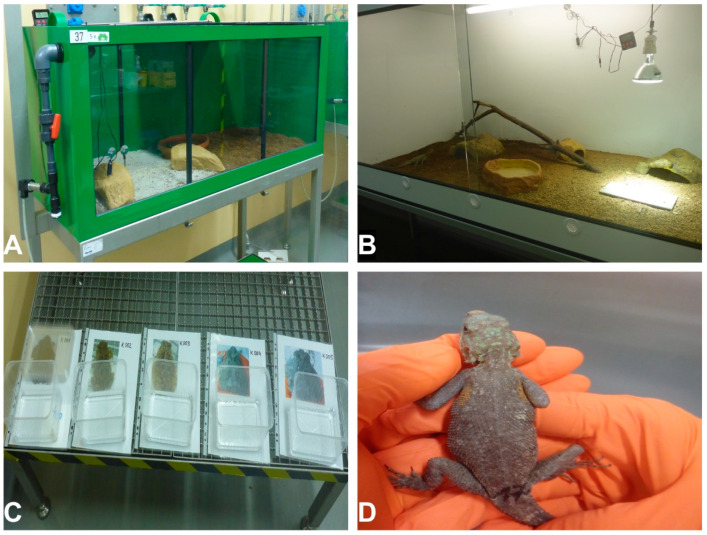
Husbandry of animals. (**A**) Husbandry of toads, (**B**) husbandry of agamas, (**C**) individual identification of toads, (**D**) individual identification of agamas.

Thirty African common toads and 32 common agamas (additionally including two reserve animals for the case of pre-infection, non-experimental induced animal fatalities, etc.) were acquired from specialized exotic pet shops. Animals were kept within the biosafety level 3 (BSL-3) containment facility of the Friedrich-Loeffler-Institut, Riems. Toads and agamas were kept in terraria (on coconut fiber and a sand–clay mixture bedding). Possibilities for bathing, climbing and hiding were provided. For agamas, UV light and a focal heat source were provided (Figure 1). Temperature and humidity were closely monitored with special herpetological hygrometers. Agamas and toads were kept at a temperature of about 25 °C and a humidity of 60–70%. Agamas additionally had a focal heat source with local temperatures of about 40 °C. The terraria were regularly humidified with aerosolized water. Water was supplied ad libitum and mealworms and crickets, regularly supplemented with the mineral and vitamin compound Korvimin^®^ (WDT, Garbsen, Germany), were offered for feeding. Toads were identified individually based on their back patterns (photographed in high resolution and pictures printed on individual ID cards for animal discrimination). Agamas were marked with numbers at their tail root (Figure 1).

Within a minimal period of seven days, animals were adapted to the local husbandry. Within this period, all toads were dewormed with an Ivermectin bath (Ivermection pour-on, Durvet, Blue springs, MO, United States) and all agamas were dewormed with an oral treatment of Fenbendazol (Panacur, Intervet, Unterschleißheim, Germany).

Prior to the infection, lymph (toads) and sera (agamas) of the animals tested serologically negative by a serum neutralization test. Subsequently, 12 African common toads and 13 common agamas were infected subcutaneously (0.2 mL in the axillary region) with the RVFV vaccine strain MP-12 with a dose of 1 × 10^5.3^ TCID_50_. Three African common toads and three common agamas were injected with the same volume of virus-free Dulbecco’s modified Eagle medium (DMEM) accordingly and served as contact controls for potential horizontal transmission (Table 1). Another 12 African common toads and 13 common agamas were infected subcutaneously (0.2 mL in the axillary region) with the RVFV strain ZH501 with a dose of 1 × 10^5^ TCID_50_. Three African common toads and three common agamas served again as contact controls and were injected with DMEM (Table 1).

After inoculation, lymph (toads) or serum (agamas) was taken at 3, 10, 16 and 21 days post-infection (dpi). Lymph from toads was drawn from the dorsal lymph sacs after hydration in a water bath. Serum from agamas was drawn from the coccygeal vein. Oral and cloacal swabs were taken at 2, 4, 7, 9, 11, 14, 16 and 18 dpi. Additionally, swabs from skin secretion of toads were taken at 2, 4, 7, 9, 11, 14, 16 and 18 dpi accordingly. At the same days, samples of the water bath were taken as environmental viral shedding controls. The animals were observed daily for behavioral and clinical anomalies (score sheet, see Appendix A Table A1). The first group of five toads and agamas (four infected animals, one non-infected control animal) was euthanized at 3 dpi, the second group at 10 dpi and the third group at 21 dpi. Animals were sedated with an intramuscular injection of Ketamin (Medistar, Ascheberg, Germany) and Xylazin (Cp-pharma, Burgdorf, Germany) and were euthanized by an intraperitoneal injection of Pentobarbital (Eutha^®^77, Pfizer animal health, Parsippany-Troy Hills, NJ, USA).

The competent authority of the Federal State of Mecklenburg Western-Pomerania approved all described animal experiments based on European Directive 2010/63/EU and the associated national regulation (reference number in Germany LALLF M-V/TSD/7221.3-1-086/16).

### 2.3. Serology

#### 2.3.1. Serum Neutralization Test

The serum neutralization test (SNT) was performed as described in the OIE Terrestrial Manual 2014 [46]. Briefly, 100 TCID_50_ of MP-12 was added to serial two-fold diluted and heat-inactivated sera or lymph. Following an incubation of 30 min at 37 °C and 5% CO_2_, 3 × 10^5^ Vero 76 cells were added to each well. Plates were incubated at 37 °C, 5% CO_2_ for 6 days. Neutralizing doses of 50% (ND_50_) were expressed as the reciprocal of the serum dilution that still inhibited >50% of the cytopathogenic effect. The lowest detectable titer of neutralizing antibodies was 1:10.

#### 2.3.2. Competition ELISA

Lymph and serum samples with sufficient volume were additionally tested with the ID Screen^®^ RVFV Competition multi-species ELISA (IDvet, Montpellier, France) according to the manufacturer’s instructions. The ELISA is based on the nucleoprotein and antibody isotypes (IgG and IgM) are indistinguishable. Although, to the authors knowledge, no previous testing of reptile serum and amphibian lymph fluid has been reported, the basic configuration of this species- and matrix-independent competitive ELISA should also make it suitable for the here tested experimental samples.

### 2.4. Detection of RVFV-Specific RNA

RNA from the swab medium, serum and lymph was extracted using the NucleoMag^®^ VET Kit (Macherey-Nagel, Düren, Germany) and King Fisher™ Flex Purification System (Thermo Fisher Scientific, Waltham, MA, USA), according to the manufacturer’s instructions. RNA of cerebrum, heart, lung, liver, spleen, kidney and small intestine was extracted using the RNeasy Mini kit (Qiagen, Hilden, Germany). As internal extraction control, an MS2 bacteriophage was added to each sample [47]. The presence of RVFV-derived RNA was verified using a quantitative real-time RT-PCR (qRT-PCR), with a detection limit of five copies per reaction [48]. A synthetic RNA calibrator was utilized for quantification [49].

### 2.5. Virus Isolation

Samples that tested positive in qRT-PCR were tested for the presence of functional virus by inoculation of Vero 76 cells or Vero E6 cells accordingly. Samples or tissue homogenates were diluted in MEM with penicillin, streptomycin and 2% fetal calve serum (FCS) and were added to 90% confluent cells. Cells were incubated for 1 h at 37 °C, 5% CO_2_. Following adsorption, fresh medium was added. All cells were incubated for seven days with daily control for cytopathogenic effects.

Cell culture supernatants of those samples were additionally double-blind-passaged on Vero cells. For each passage, the cell culture supernatants were adsorbed on Vero cell monolayers for 1 h at 37 °C, 5% CO_2_, overlaid with medium and incubated at 37 °C for 7 days. At 7 dpi, cell culture supernatants were transferred to the next passage. RNA was isolated from all cell culture supernatants and a qRT-PCR was performed to validate the presence of RVFV. For this purpose, RNA was extracted using the NucleoMag^®^ VET Kit (Macherey-Nagel) and King Fisher™ Flex Purification System (Thermo Fisher Scientific).

### 2.6. Necropsy, Histopathological and Immunohistopathological Examination

Necropsies were performed according to standard procedures under BSL-3 conditions. Specimens of brain, heart, lungs, liver, spleen, kidneys and variably also intestine were fixed in 4% neutral buffered formaldehyde, processed, embedded in paraffin wax, sectioned at 2–4 µm thickness and stained with hematoxylin and eosin.

Immunohistology was performed on the following sections for the ZH501-infected agama with the avidin–biotin–peroxidase complex method (Elite PK6100 kit; Vector Laboratories, Burlingame, CA, USA), citric buffer antigen retrieval, 3-amino-9-ethylcarbazole (Dako, Glostrup, Denmark) as chromogen and hematoxylin counterstain. A mouse hybridoma supernatant containing IgG1 directed against the Gc protein of RVFV (clone9A9) was used as primary antibody in a dilution of 1:1000. MP-12-infected and -uninfected Vero 76 cell pellets served as positive and negative controls, respectively. Further negative controls consisted of replacement of the primary antibody by tris buffered saline on serial sections.

## 3. Results

### 3.1. Clinical Assessment

**Figure 2:**
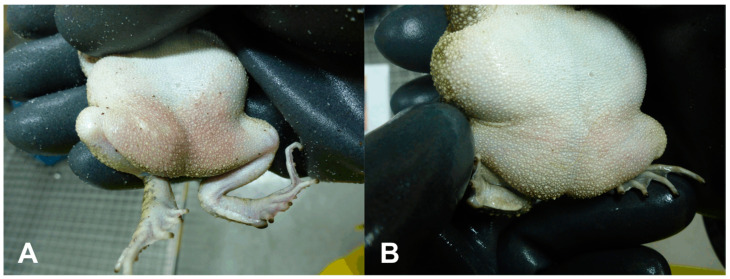
Hyperemia in ZH501-infected toads. (**A**) ZH501-infected toad (#K28-Z) at 16 dpi with mild hyperemia, apparent through reddened skin at the lower abdomen and hind legs; (**B**) non-infected toad (#K30-Z) at 16 dpi.

No significant clinical deviations could be observed in both MP-12- and ZH501-infected toads and agamas. Only in ZH501-infected toads, a mild hyperemia was observed starting at 8 dpi (in four out of eight infected toads), which might display an activation of enhanced immune defense within the circulation system (Figure 2).

### 3.2. Serology

**Figure 3:**
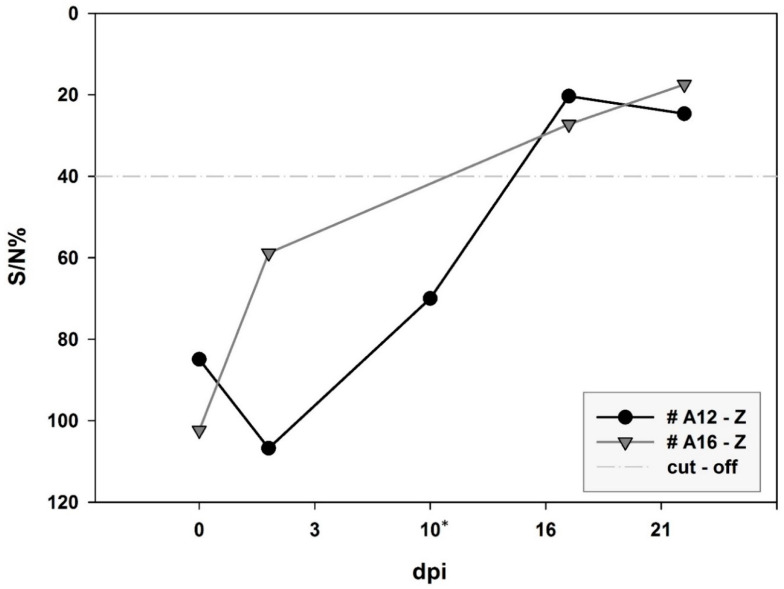
ID Vet Competition ELISA. * No serum was available from #A16-Z at 10 dpi.

Neutralizing antibodies were only detected in two MP-12-infected agamas (#A12-M and #A14-M) at 16 and 21 dpi. Titers were at the lowest detectable levels (ND_50_ of 1:10). No neutralizing antibodies were detected in MP-12- and ZH501-infected toads. Sera from ZH501-infected agamas caused massive fungal contaminations of the cell cultures. All agama sera were therefore run in the IDvet Competition ELISA, but only two ZH501-infected agamas (#A12-Z and #A16-Z) displayed a seroconversion against RVFV NP, starting at 16 dpi (Figure 3). All other samples were negative.

### 3.3. Detection of Viral RNA and Virus Isolation

#### 3.3.1. Toads Infected with MP-12

In all collected lymph, swab and tissue samples, no RVFV-derived genomes could be detected.

#### 3.3.2. Toads Infected with ZH501

No RVFV-derived genomes were detected in qRT-PCR in swabs and tissues of the ZH501-infected toads. Only in the lymph of two infected toads at 3 dpi, low signals were revealed. Although Ct values decreased during the first passage, no viral RNA was detected in the second passage and no replicating viruses were rescued (Table 2A).

#### 3.3.3. Agamas Infected with MP-12

No RVFV-derived genomes were detected in swabs and serum of MP-12-infected agamas. Occasionally, evidence of RVFV-specific RNA was found in different tissues (liver, lungs, brain and kidney) at 3 and 10 dpi, but rather at a very low level. Additionally, no increases in RNA levels were observed by passaging and no replicating viruses were rescued (Table 2B).

#### 3.3.4. Agamas Infected with ZH501

No RVFV-derived genomes were recovered from serum of ZH501-infected agamas. All spleens collected from infected animals sacrificed at 3 dpi were positive in qRT-PCR. One sample (#A17) showed a clear decrease in Ct during passaging and a replicating virus was rescued. Furthermore, low-level signals were observed in oral swabs of three agamas at different time points. However, no decreases in Ct values were observed during passaging and no replicating viruses were rescued from these samples (Table 2C).

### 3.4. Necropsy, Histopathological and Immunohistopathological Examination

#### 3.4.1. Toads Infected with MP-12 and ZH501

None of the MP-12- and ZH501-infected or contact control toads displayed macroscopic findings typical for RVF, such as multifocal to diffuse necrosis. Immunohistology was omitted for the toads due to the generally low to negative qRT-PCR results of the post-mortal specimen and the lower sensitivity of the immunohistology. Pathological findings unrelated to the challenge studies were quite frequently observed in the toads (see Appendix A).

#### 3.4.2. Agamas Infected with MP-12 and ZH501

None of the MP-12- and ZH501-infected or contact control agama displayed macroscopic findings typical for RVF, such as multifocal to diffuse necrosis. Immunohistology revealed no RVFV antigen within the brain, heart, lungs, liver, spleen, kidney, pancreas, intestine and gonads of ZH501-infected agamas. Histopathological and immunohistological work-up was omitted for the MP-12-infected agamas due to the lack of clinical signs of RVF, lack of macroscopic lesions and the generally low to negative qRT-PCR results of the post-mortal specimens and the lower sensitivity of the immunohistology. Pathological background findings of agamas are described in Appendix A.

## 4. Discussion

For decades, finding a reservoir host has been one of the open questions in RVF virus ecology. As field and in vitro study data indicated a potential role of amphibians and reptiles in the enzootic cycle of RVFV, we have addressed this question by an experimental in vivo challenge approach.

African common toads (*Amietophrynus regularis*) and common agamas (*Agama agama*) were exemplarily chosen as representative species, due to their wide distribution over the African continent [50,51] and the plausibility of interaction with RVFV competent vectors. Our experimental data show that toads are resistant to a subcutaneous infection with RVFV strains MP-12 and ZH501. The MP-12-infected toads showed no seroconversion and no viremia or shedding of virus. Moreover, all tissues remained negative in qRT-PCR. Likewise, no seroconversion was detected in ZH501-infected toads. RVFV-derived genomes were found in the lymph of two toads at 3 dpi, albeit genome copy numbers were very low (<one copy/µL) and virus could not be re-isolated (by double-blind cell culture passages). No shedding was observed for ZH501-infected toads and no RVFV-derived genome was detected in the tissues.

For ranavirus (FV3) infection, it has been shown that the virus clearance initiated by CD8+ T-cells starts at 6 dpi [52]. As no virus was detected in any target tissues in the infected toads at 3 dpi, it seems that the virus was cleared previously. As amphibian-derived cell cultures can be infected productively with RVFV in principle [42], it may rather be innate defense mechanisms such as antimicrobial peptides (AMPs) or interferons, which have caused the initial virus control and clearance. For FV3 [53,54,55], dengue virus [56], influenza viruses [57], herpes simplex virus [58] and HIV [59], it is assumed that AMPs play an important role in inactivating viruses at their entry sites and are also controlling the generalization of the infections already before adaptive immune responses would take over. Although the activation of the innate immune response is probably of utmost importance for the clearance of infection, a demonstration of its activation after the experimental infection of toads and agamas is unfortunately not feasible due to the lack of species-specific reagents. Another aspect that may have supported the refractory infection is the observed parasitic infection, as previous studies found that a trematode infection is beneficial for hosts exposed to viral infection, potentially through cross-reactive immunity [60].

For both MP-12- and ZH501-infected toads, no seroconversion was detected. These results are in accordance with previous data, since experimental infection of amphibians with FV3 did not induce a generation of specific IgM or IgY antibodies for up to a month after infection [61] or even after the second infection with the pathogen [62]. It is generally known that amphibians rely primarily on mechanisms of nonspecific innate immune responses, while the serological responses are rather of subordinate importance [63].

Only few RVFV MP-12-infected agamas (*n* = 2 out of 13) developed a faint neutralizing antibody response (ND_50_ of 1:10). Neither viremia nor shedding was observed in the animals. However, viral RNA was found in multiple tissues at 3 and 10 dpi, but no replicative virus was re-isolated. Likewise, only 2 out of 13 RVFV ZH501-infected agamas seroconverted. Signals were observed in the IDvet Competition ELISA; however, a verification by SNT was not possible due to a massive fungal contamination of all sera. Viremia was not detected in any tested sera, but RVFV-derived genomes were found in three oral swabs from 7, 9 and 14 dpi. Virus isolation attempts were unsuccessful. In contrast, an RVFV-specific genome was consistently detected in the spleen of ZH501-infected agamas euthanized at 3 dpi and even a virus isolate was retrieved from one sample (#A17-Z; 3 dpi).

The almost absent serological response in agamas may also be caused by the slow humoral response that is common in reptiles. It has been reported that substantial antibody levels are reached after vaccinations only as late as six to eight weeks. Reptiles rely more heavily on alternative antibody responses by natural antibodies that are released spontaneously in the absence of antigen stimulation [64]. Additionally, other experimental in vivo studies have demonstrated that not all animals seroconvert collectively [26].

The results of this study indicate different RVFV susceptibilities of African common toads and common agamas. While the study supposes that toads do not seem to play a role as a reservoir for RVFV at all, a role of agamas, albeit of minor importance, cannot be finally excluded. Other authors also observed significant differences comparing viral susceptibility of amphibians and reptiles [65]. Additionally, previous investigations found high diversity between different species of the same class, showing absolute resistance in one species compared to susceptibility in another [26,66]. Consequently, our results are not easily true for other amphibian and reptilian species. Additionally, perspective experiments should evaluate potential differences when using a challenge virus that is grown in insect cells, as it more closely mimics nature, and sometimes results in different pathogenicity. Other than that, an increase in viral dose might cause different manifestations and courses of infection and should be a focus of future experiments. Furthermore, changes in the temperature of terraria might also influence the outcome of infection and should be considered for following studies. Further in vitro and field study data are needed to conclusively determine the role of amphibians and reptiles in the RVFV infection cycle. Serological investigations are an easy and valuable tool, but since seroconversion was detected only irregularly and at very low levels, such studies may produce only unreliable results.

## 5. Conclusions

In conclusion, the results of this study indicate that despite their enormous biological potential, toads do not play a role in the RVFV ecology. The same may also be true for agamas, albeit a subordinate role may be possible. However, findings cannot be applied for the whole class of amphibians and reptiles, thus their definite role as potential reservoir hosts for RVFV needs to be investigated prospectively.

## Figures and Tables

**Table 1:** Individual animal identification and assignment.

Toads			Agamas		
ID	Infected with	Euthanized at (dpi)	ID	Infected with	Euthanized at (dpi)
#K01-M	MP-12	3	#A01-M	MP-12	3
#K02-M	MP-12	3	#A03-M	MP-12	3
#K03-M	MP-12	3	#A04-M	MP-12	3
#K04-M	MP-12	3	#A18-M	MP-12	3
#K05-M	Mock	3	#A05-M	Mock	3
#K06-M	MP-12	10	#A06-M	MP-12	10
#K07-M	MP-12	10	#A07-M	MP-12	10
#K08-M	MP-12	10	#A08-M	MP-12	10
#K09-M	MP-12	10	#A09-M	MP-12	10
#K10-M	Mock	10	#A10-M	Mock	10
#K11-M	MP-12	21	#A11-M	MP-12	21
#K12-M	MP-12	21	#A12-M	MP-12	21
#K13-M	MP-12	21	#A13-M	MP-12	21
#K14-M	MP-12	21	#A14-M	MP-12	21
#K15-M	Mock	21	#A15-M	Mock	21
			#A16-M *	MP-12	21
#K16-Z	ZH501	3	#A01-Z	ZH501	3
#K17-Z	ZH501	3	#A03-Z	ZH501	3
#K18-Z	ZH501	3	#A04-Z	ZH501	3
#K19-Z	ZH501	3	#A17-Z	ZH501	3
#K20-Z	Mock	3	#A05-Z	Mock	3
#K21-Z	ZH501	10	#A06-Z	ZH501	10
#K22-Z	ZH501	10	#A07-Z	ZH501	10
#K23-Z	ZH501	10	#A08-Z	ZH501	10
#K24-Z	ZH501	10	#A09-Z	ZH501	10
#K25-Z	Mock	10	#A10-Z	Mock	10
#K26-Z	ZH501	21	#A11-Z	ZH501	21
#K27-Z	ZH501	21	#A12-Z	ZH501	21
#K28-Z	ZH501	21	#A13-Z	ZH501	21
#K29-Z	ZH501	21	#A15-Z	Mock	21
#K30-Z	Mock	21	#A16-Z	ZH501	21
			#A18-Z *	ZH501	21

* reserve animal.

**Table 2:** Detection of viral RNA.

(**A**) ZH501-Infected Toads					
Sample *	**Animal**	**dpi**	**qRT** **-PCR—Ct Values (Copies/µL RNA)**		
			**Original**	**1st Passage**	**2nd Passage**
Lymph	#K026-Z	3	39 (<1)	37 (<1)	-
Lymph	#K028-Z	3	41 (<1)	38 (<1)	-
(**B**) MP-12-Infected Agamas					
Sample *	**Animal**	**dpi**	**qRT** **-PCR—Ct Values (Copies/µL RNA)**		
			**Original**	**1st Passage**	**2nd Passage**
Liver	#A03-M	3	35 (25)	-	-
Lungs	#A03-M	3	38 (4)	-	-
Brain	#A04-M	3	35 (25)	-	-
Kidney	#A06-M	10	39 (3)	-	-
(**C**) ZH501-Infected Agamas					
Sample *	**Animal**	**dpi**	**qRT** **-PCR—Ct Values (Copies/µL RNA)**		
			**Original**	**1st Passage**	**2nd Passage**
Spleen	#A01-Z	3	37 (3)	-	-
Spleen	#A03-Z	3	38 (1)	-	-
Spleen	#A04-Z	3	36 (7)	-	-
Spleen	#A17-Z	3	33 (51)	31 (154)	21 (813,000)
Oral swab	#A12-Z	7	39 (1)	-	-
Oral swab	#A16-Z	9	39 (1)	-	-
Oral swab	#A18-Z	14	39 (1)	-	-

* Only qRT-PCR positive samples are displayed. All other samples were tested negative.

## Appendix A

Pathological examination—Background findings.

Toads infected with MP-12 and ZH501.

Macroscopic background findings unrelated to group and time point included ascites (*n* = 12 out of 30), focal or multifocal hepatic masses (*n* = 9 out of 30), anemia (*n* = 2 out of 30), emaciation (*n* = 2 out of 30), focal or multifocal serosal mass (N = 2 out of 30) and trematodiasis of the urinary bladder (*n* = 1 out of 30; morphology consistent with *Polystoma* spp.).

Over and above, histopathology revealed chronic, proliferative and/or granulomatous cholangitis and/or hepatitis with undefined etiology (*n* = 6 out of 30), chronic, proliferative and/or granulomatous cholangitis and/or hepatitis with adult trematodes (*n* = 5 out of 30; morphology consistent with *Glypthelmins* spp.), granulomatous hepatic subcapsular masses with cestodes (*n* = 2 out of 30), granulomatous intrahepatic masses with nematodes (*n* = 1 out of 30), chronic lymphohistioplasmacytic and/or granulomatous interstitial nephritis (*n* = 9 out of 30), focal or multifocal granulomatous pneumonia (*n* = 2 out of 30), focal lymphohistiocytic myocarditis (*n* = 2 out of 30) and focal granulomatous splenitis (*n* = 1 out of 30).

Agamas infected with MP-12 and ZH501.

Macroscopic background findings unrelated to group and time point included arthropod ectoparasites (mites; *n* = 3 out of 32) and intestinal cestodiasis (*n* = 2 out of 32).

Over and above, histopathology of the ZH501-infected agama revealed apicomplexian, intraepithelial schizonts and intraluminal oocysts within the bile ducts (morphology consistent with *Choleoeimeria* spp.; *n* = 11 out of 13), and intracholangiolar trematodes (*n* = 1 out of 13) with variable associated inflammation, lymphohistiocytic interstitial nephritis (*n* = 3 out of 13), intraluminal cestodes within the intestine (*n* = 3 out of 13), apicomplexian intramucosal gamonts and intraluminal oocysts within the intestine (morphology consistent with *Eimeria* spp.; *n* = 2 out of 13).

**Table A1:** Score sheet.

Category	Findings	Score
General condition	Not disturbed: showing species-specific behavior	0
	Mildly disturbed: listless, restless	5
	Moderately disturbed: apathetic	10
	Highly disturbed: comatoseWarning: toads like to bury themselves under the sand and do rest like that, do not misunderstand it as apathetic behavior. The behavior should ideally be observed after putting them back to the terraria after handling.	20
Behavior	Normal: sleeping, reaction to stimuli, curiosity, social contact to other animals	0
	Mild deviation from normal behavior	1
	Unusual behavior, limited motoric or hyperkinetic	5
	Self-isolation, lethargy; significant hyperkinetic	10
	Auto-aggression	20
State of care	Smooth, intact skin (apart from moltings); clean body orifices; clear and shining eyes	0
	Small defects in the skin	5
	Neglected body orifices; cloudy eyes; increased tone of muscles	10
	Dirty skin with inflammation and/or injuries; sticky body orifices; unusual body position; cloudy eyes; high tone of muscles	20
Nutritional status (body weight)	Unaffected or increasing	0
	Loss of body weight about 10% of original weight	5
	Loss of body weight about 15% of original weight	10
	Loss of body weight about 20% of original weightWarning: Toads tend to excrete massive volumes of urine as a defense mechanism. Sudden losses of body weights have to observed for a period of three days.	20
Clinical symptoms	Normal breathing, mucous membranes are well supplied with blood	0
	Mild deviations of the normal situation	1
	Increased breathing after stress	5
	Increased breathing during rest; pale mucous membranes; sporadic bleedings; disturbance in coordination	10
	Heavy breathing; nasal and conjunctival discharge; cyanotic mucous membranes; disturbances of the central nervous system; generalized bleeding	20

**Table A2:** Abort criteria.

Additive Score	Grade of Stress	Assessment and Measures That Have to be Taken
0 to 2	0	No stress No measures
6 to 25	1	Low level of stress Careful further observation
26 to 50	2	Moderate level of stress Initiate veterinary care (for example, analgesia, etc.) Careful further observation Consultation of animal welfare officer
>50	3	High level of stress Immediate euthanasia of the animal
